# Low Health System Performance, Indigenous Status and Antivenom Underdosage Correlate with Spider Envenoming Severity in the Remote Brazilian Amazon

**DOI:** 10.1371/journal.pone.0156386

**Published:** 2016-05-26

**Authors:** Vanderson Souza Sampaio, André Alexandre Gomes, Iran Mendonça Silva, Jacqueline Sachett, Luiz Carlos Lima Ferreira, Sâmella Oliveira, Meritxell Sabidò, Hipócrates Chalkidis, Maria Graças Vale Barbosa Guerra, Jorge Luis Salinas, Fan Hui Wen, Marcus Vinícius Guimarães Lacerda, Wuelton Marcelo Monteiro

**Affiliations:** 1 Diretoria de Ensino e Pesquisa, Fundação de Medicina Tropical Dr. Heitor Vieira Dourado, Manaus, Brazil; 2 Escola Superior de Ciências da Saúde, Universidade do Estado do Amazonas, Manaus, Brazil; 3 Núcleo de Sistemas de Informação, Fundação de Vigilância em Saúde do Amazonas, Manaus, Brazil; 4 Department of Medical Sciences, Faculty of Medicine, Universitat de Girona, Catalunya, Spain; 5 Curso de Ciências Biológicas, Faculdades Integradas do Tapajós, Santarém, Pará, Brazil; 6 Department of Medicine, Emory University, Atlanta, Georgia, United States of America; 7 Instituto Butantan, Secretaria de Estado da Saúde de São Paulo, São Paulo, Brazil; 8 Instituto de Pesquisas Leônidas & Maria Deane, FIOCRUZ, Manaus, Brazil; Universidad de Costa Rica, COSTA RICA

## Abstract

**Background:**

A better knowledge of the burden and risk factors associated with severity due to spider bites would lead to improved management with a reduction of sequelae usually seen for this neglected health problem, and would ensure proper use of antivenoms in remote localities in the Brazilian Amazon. The aim of this study was to analyze the profile of spider bites reported in the state of Amazonas in the Western Brazilian Amazon, and to investigate potential risk factors associated with severity of envenomation.

**Methodology/Principal Findings:**

We used a case-control study in order to identify factors associated with spider bite severity in the Western Brazilian Amazon from 2007 to 2014. Patients evolving to any severity criteria were considered cases and those with non-severe bites were included in the control group. All variables were retrieved from the official Brazilian reporting systems. Socioeconomical and environmental components were also included in a multivariable analysis in order to identify ecological determinants of incidence and severity. A total of 1,181 spider bites were recorded, resulting in an incidence of 4 cases per 100,000 person/year. Most of the spider bites occurred in males (65.8%). Bites mostly occurred in rural areas (59.5%). The most affected age group was between 16 and 45 years old (50.9%). A proportion of 39.7% of the bites were related to work activities. Antivenom was prescribed to 39% of the patients. Envenomings recorded from urban areas [Odds ratio (OR) = 0.40 (95%CI = 0.30–0.71; p<0.001)] and living in a municipality with a mean health system performance index (MHSPI >median [OR = 0.64 (95%CI = 0.39–0.75; p<0.001)] were independently associated with decreased risk of severity. Work related accidents [OR = 2.09 (95%CI = 1.49–2.94; p<0.001)], Indigenous status [OR = 2.15 (95%CI = 1.19–3.86; p = 0.011)] and living in a municipality located >300 km away from the state capital Manaus [OR = 1.90 (95%CI = 1.28–2.40; p<0.001)] were independently associated with a risk of severity. Living in a municipality located >300 km away from the state capital Manaus [OR = 1.53 (95%CI = 1.15–2.02; p = 0.003)] and living in a municipality with a MHSPI <median [OR = 1.91 (95%CI = 1.28–2.47; p = 0.002)] increased the odds of antivenom underdosage.

**Conclusions:**

Spider bites is prevalent across the study region with a higher incidence in the rainy season in rural areas. Spider bites can be painful and lead to local manifestations but rarely result in life-threatening envenoming. Major local complications were dermonecrosis and secondary infection in cases diagnosed as *Loxosceles* bites. Based on the correlations shown here, envenomings occurring in remote rural areas, Indigenous status and living in a municipality located >300 km away from the state capital Manaus could be contributing factors to higher severity of spider envenomings in this area, as well as to antivenom underdosage.

## Introduction

Spider bites are common in most parts of the world, but in general cause only minimal effects [1,2]. However, the epidemiology of this neglected condition differs throughout the world and its burden is often poorly estimated. There are few official surveillance systems for spider bites reporting annual or periodic epidemiological data on spider bites across the world. The American Association of Poison Control Centers [3], the Australian Poison Information Center [4] and the national surveillance system from the Brazilian Ministry of Health [5] provide data on spider bites at a national level by using different surveillance methods. In Brazil envenomations, among which is included spider bites, are notifiable injuries and that must be recorded in the Brazilian Notifiable Diseases Surveillance System [*Sistema de Informação de Agravos de Notificação* (SINAN)]. A total of 270,885 cases spider bites were recorded by the Brazilian surveillance system from 2000 to 2013, with a notable increasing trend in the period, from 3,257 cases in 2000 (1.9 cases/100,000 inhabitants) to 27,125 in 2013 (14.6 cases/100,000 inhabitants) [6]. The incidence is higher in Southern Brazil, with a rate of 60.5 cases/100,000 inhabitants in 2013. In the same year, there were 5.2 cases per 100,000 in the Brazilian Amazon [7]. The number of cases detected officially in the Amazon is probably much lower than the real number due to the difficulty for riverine and indigenous populations to reach health centers for treatment of venomous animal injuries. Indeed, an epidemiological survey carried out in the state of Acre, Western Brazilian Amazon, found that 7% of tappers and 11% of Amerindians were bitten by spiders at least once in their lifetime [8]. Moreover, the lethality rate from spider bites in the Amazon (0.2%) is almost seven times higher than in Southern country (0.03%) [9].

Spider bite envenoming is caused by the inoculation of spider toxins through the inoculum apparatus and may cause local and systemic changes. Latrodectism and loxoscelism are the most important clinical syndromes resulting from spider bites [10]. Latrodectism results from bites by widow spiders (*Latrodectus* spp) and causes local, regional, or generalized pain associated with non-specific symptoms and autonomic effects [10–12]. Loxoscelism is caused by *Loxosceles* spp, and the cutaneous form manifests as pain and erythema that can develop into a necrotic ulcer. Systemic loxoscelism is characterised by intravascular haemolysis and renal failure [10,13–15].

In Brazil, the spiders causing more serious human accidents belong to the genera *Phoneutria*, *Loxosceles* and *Latrodectus* [5,16,17]. Loxoscelism is the most widely spread spider bite syndrome in the country, occurring with higher frequency from October to March, while most of the phoneutrism cases are recorded from January to May, with higher incidence in the South [5]. Latrodectism presents low incidence, with records mostly from Minas Gerais, São Paulo, Bahia and Santa Catarina states [5]. Two types of spider antivenoms are currently available in Brazil: the polyvalent antivenom against *Loxosceles* and *Phoneutria* spiders and the *Tityus* scorpion, produced from *Loxosceles gaucho*, *Phoneutria nigriventer* and *Tityus serrulatus* pool, and the anti-*Loxosceles* antivenom, produced from *Loxosceles gaucho*, *L*. *intermedia* and *L*. *laeta* pool [5]. However, the possible interspecific venom variation associated with the geographical distribution of spider species may affect the effectiveness of therapeutic antivenoms against the Amazon spider venoms.

**Fig 1:**
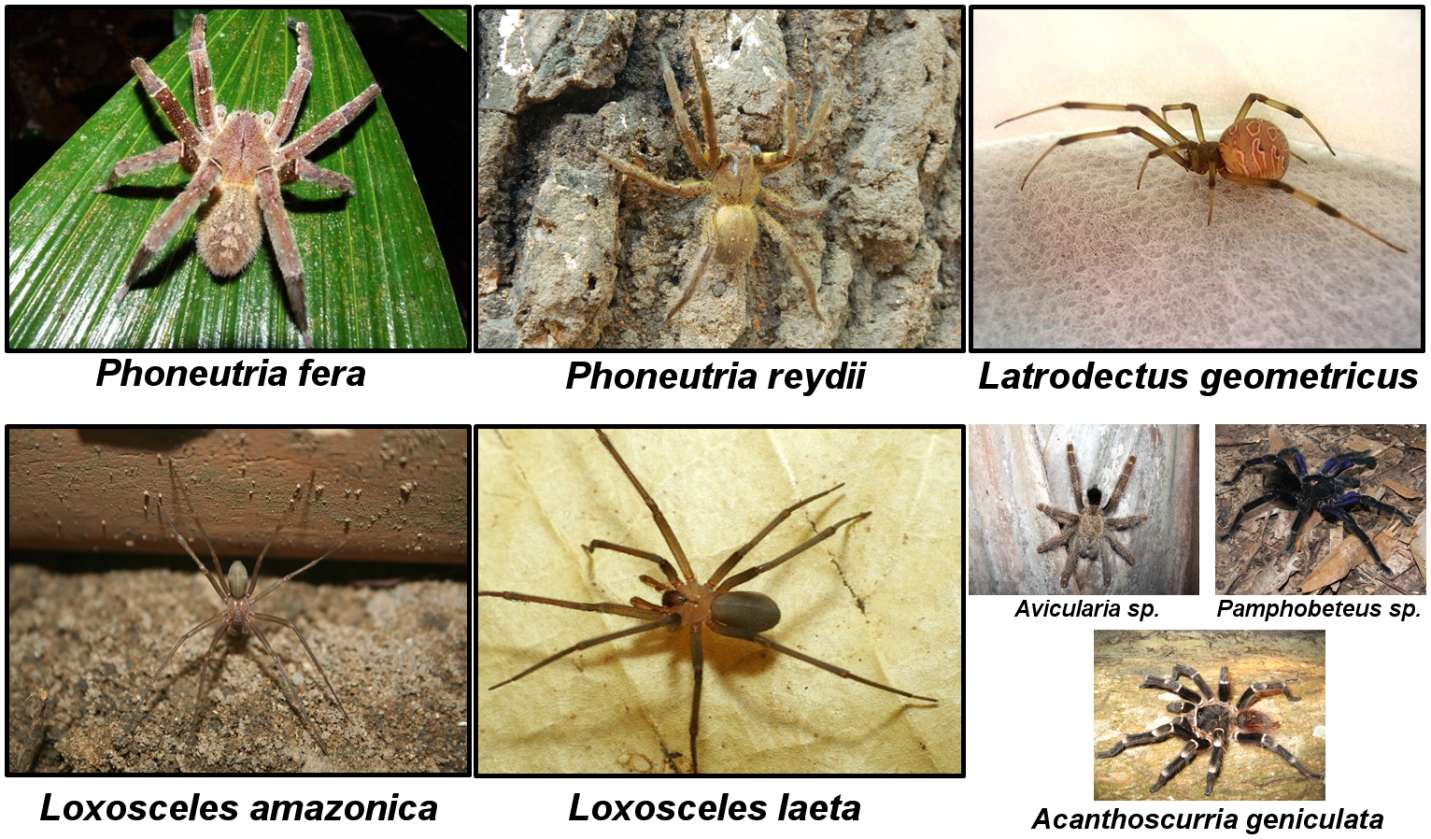
Main spider species of medical significance in the Brazilian Amazon. *Phoneutria fera* (A) and *Phoneutria reydii* (B), commonly known as Brazilian wandering spiders, armed spiders ("armadeiras", as they are known in Brazilian Portuguese), or banana spiders, are a genus of aggressive spiders of the Ctenidae family, with potential medical significance to humans due to their neurotoxic venom. *Phoneutria fera* is a species largely distributed in Brazil, Ecuador, Peru and in the Guyanas. *Phoneutria reydii* is found in Brazil, Venezuela and the Guyanas. *Latrodectus geometricus* (C), commonly known as the brown widow, brown button spider, geometric button spider or gray widow ("viúva negra" or "aranha preta", as is known in Brazil), is a medium spider of the family Theridiidae that belongs to a species group of medical interest due to its neurotoxic venom effect on human health. Probably native from Africa, the species is largely distributed in most American countries. *Loxosceles amazonica* (D) and *Loxosceles laeta* (E), known as recluse spiders, fiddle-back, violin spiders or reapers (commonly known as "aranha marrom" in Brazil), are non-aggressive venomous spiders belonging to the family Sicariidae, which can induce a variety of clinical symptoms, including dermonecrosis, thrombosis, vascular leakage, haemolysis and persistent inflammation. *Loxosceles amazonica* is found in Northern and Northeastern Brazilian regions and *Loxosceles laeta* is found all over South America (Chile, Peru, Ecuador, Argentina, Brazil, Ecuador and Colombia) and in Central America (Guatemala and Honduras). Three specimens of spiders (F) belonging to the Theraphosidae (called "caranguejeiras" and "tarântulas" in Portuguese) with limited medical importance. Although tarantulas are venomous and some bites cause local pain, these generally evolve without systemic manifestations.

The contribution of different spider species responsible for spider bites to the overall burden needs to be assessed through population- and hospital-based studies. Usually the agent is not identified and the diagnosis is based mostly on clinical and epidemiological features, leading to a limitation in monitoring and properly treating this health problem [5]. Based on fauna distribution data, *Phoneutria fera* and *Phoneutria reidyi* likely represent the major species leading to phoneutrism in the region [17]. *Loxosceles amazonica* and *Loxosceles laeta* are thought to be the major agents of loxoscelism in the Brazilian Amazon [17,18]. Among *Latrodectus* species, *Latrodectus geometricus*, the most widely species found in Brazil, is thought to be the major causative of envenomings [17]. The data on the biology and behavior of these spider species in the Amazon biome remain unclear, especially in the artificial ecotopes with possible differences in spider species composition. Fig 1 shows main species of spiders responsible for bites in the Brazilian Amazon.

A better knowledge of severity due to spider bites would lead to improved management, with expected reduction of sequelae and lethality rates in remote localities in the Brazilian Amazon. The aim of this study was to analyze the profile of spider bites reported in the state of Amazonas in the Western Brazilian Amazon, and to investigate potential risk factors for associated severity.

## Methods

### Study area and population

The State of Amazonas is located in the Western Brazilian Amazon, comprising an area of 1,570,946.8 km^2^, with 62 municipalities. The estimated population of the state was 3,807,921 inhabitants in 2010, with 74.2% living in urban zones and 25.8% in rural areas. We included all spider bite cases in the state of Amazonas compulsorily recorded by SINAN from January 1, 2007 to December 31, 2014, based on data report forms used in the investigation and follow-up of cases. Spider bite antivenom is provided free of charge only by the Brazilian Government, linked to SINAN recording, and is not available from the private sector.

### Exposures and outcome definitions

Reporting on spider bite’s severity grading and outcomes (discharge or death) is required by SINAN and entered since 2007 by healthcare providers at the time of case notification (which often happens after discharge) in the Amazonas state. The variables analyzed were sex, age, anatomical region of the bite, area of occurrence (rural or urban), work-related injury, time elapsed between the bite and medical assistance, suspected spider genus (*Phoneutria*, *Loxosceles or Latrodectus*), severity grading (mild, moderate or severe), outcome (discharge or death), clinical features and antivenom administration data. All variables were checked by two independent researchers before analysis and further investigated for possible association with severity as dependent variables.

Clinical severity of spider bites in this work was classified according to the Brazilian Health Ministry guidelines [5], as presented in Table 1.

**Table 1:** Brazilian Ministry of Health clinical classification of spider bites.

Case classification	Signals and symptoms	Antivenon recommendation
**Mild**	Local pain, edema, erythema, sweating and piloerection for *Phoneutria* bites and absence of systemic manifestations with identification of the spider for *Loxosceles* bites.	Not recommended
**Moderate**	Intense local pain, sweating, occasional vomiting, psychomotor restlessness and arterial hypertension are found in moderate *Phoneutria* bites; moderate *Phoneutria* bites are treated with 2–4 vials of the polyvalent antivenom against Loxosceles and Phoneutria spiders and the *Tityus* scorpion (LPT antivenom). Moderate *Loxosceles* bites present suggestive features regardless spider identification, nonspecific systemic manifestations (exanthema, fever), with absence of hemolysis	5 vials of LPT or anti-*Loxosceles* (AL) antivenom
**Severe**	Life-threatening spider bites, with profuse sweating, drooling, lavish vomiting, priapism, shock and/or acute lung edema for *Phoneutria* bites, and characteristic lesion plus clinical manifestations and/or laboratory evidence of intravascular hemolysis for *Loxosceles*.	i) Severe *Phoneutria*: 5–10 vials of LPT antivenom ii) Severe *Loxosceles*: 10 vials of LPT or AL antivenom.

Latrodectism is scarcely seen in Brazil, including the Amazon, and thus lacks clinical grading by the Brazilian Health Ministry guidelines. There is no anti-*Latrodectus* antivenom available in Brazil. Local bite-site pain, regional or radiating pain and local or regional diaphoresis are the major signs of latrodectism [10]. Systemic envenomation includes non-specific symptoms such as nausea, vomiting, headache, fatigue, muscle fasciculation, patchy localized paralysis, priapism, arterial hypertension, tachycardia and abdominal pain and rigidity [5,10]. In this study, *Latrodectus* envenomings presenting only with local signs were classified as mild and those presenting systemic envenomation were classified as moderate/severe.

In order to identify factors associated with spider bite severity, a case-control study was used wherein patients evolving to severe or moderate clinical picture were classified as cases and those with mild bites were included as controls. Cases without clinical classification were not included in the final analysis.

### Sociodemographical and environmental indicators

Data on the populations living in municipalities in Amazonas were obtained from the 2010 Census and the intercensus projections developed by the IBGE [19].

Fourteen socioeconomical components and subcomponents were evaluated: (1) Mean Municipal Human Development Index (MMHDI), which is the geometric mean of the indices of the dimensions level of education (EMHDI), longevity (LMHDI) and income (IMHDI), with equal weights, in addition to the three individual dimensions; (2) Gross Municipal Product; (3) Mean Health System Performance Index (MHSPI), which is composed of access (MHSPI Access) and effectiveness (MHSPI Effectiveness) subcomponents; (4) Poverty Rate, the percentage of individuals with a per capita household income less than half the minimum wage in that period; (5) Average Household Income Per Capita, which is the ratio between the sum of the income of all individuals living in permanent private households and the total number of individuals; (6) Income Ratio, which compares the average per capita income of individuals belonging to the richest fifth of this distribution with the average per capita income of individuals belonging to the poorest fifth; (7) Unemployment Rate of the population aged ≥10 years, which is the percentage of the economically active population (EAP) in this age group that was unemployed; (8) Straight-line distance from the Amazonas state capital Manaus, in kilometers, to the municipality seat; and (9) Rural Occupation Rate of the EAP that was working in rural activities. These indicators were obtained from the United Nations Development Programme and from the Brazilian System of Health [20,21].

The three environmental components studied were (1) Percentage of areas under the influence of watercourses in 2010; (2) average annual deforestation increment between 2007 and 2014; (3) average annual deforested area in the municipalities between 2007 and 2014, which was measured by the average annual percentage of the municipal area that lost forest vegetation [22].

### Data analysis

To calculate the average annual incidence rates, all new cases of spider bites reported during the study period were added, divided by the sum of the estimated population for each year, and multiplied by 100,000. A map was created with the software ArcMap 10.1 in ArcGIS 10.1 (ESRI, USA) using estimates of the mean incidence by municipality. To study the relationship between the above mentioned sociodemographical and environmental indicators and spider bites incidence, a multiple linear regression model was used. Collinearity and the interaction between the explanatory variables in the analysis that led to the final multiple regression model were appreciated. Correlation between variables was checked. For the final model, only the variables associated with the outcome at a significance level of 0.20 using the *backward stepwise* technique were selected. Proportions of severe cases were compared by Chi-square test (corrected by Fisher's test if necessary), using individual characteristics from SINAN database and socioeconomical features of the residence municipality as dependente variables; differences were considered statistically significant for p<0.05. The crude *Odds Ratio* (OR) with its respective 95% Confidence Interval (95%CI) was determined considering severity as the dependent variables. Association between time until medical assistance and distance to the capital was assessed by the Chi-square test. A logistic regression was used for the multivariable analyses and the adjusted *Odds Ratios* with 95%CI were also calculated separately for SINAN and socioeconomical variables. All variables associated with the outcomes at a significance level of p<0.20 in the univariable analysis were included in the multivariable analysis. Statistical significance was considered if p<0.05 in the Hosmer-Lemeshow goodness-of-fit test. The statistical analyses were performed using STATA statistical package (version 13).

### Ethical clearance

This study was approved by the Ethics Review Board (ERB) of the *Fundação de Medicina Tropical Dr*. *Heitor Vieira Dourado* (approval number 100832/2014). All data were anonymously analyzed, and therefore a waiver of informed consent was given.

## Results

### Descriptive characteristics of spider bites

According to the official reporting system, 1,181 spider bites were recorded in the Amazonas State from 2007 to 2014. Comprehensive recording of most relevant variables was considered good (>80%). Most of the spider bites occurred in males (777 cases; 65.8%) and 59.5% were reported in rural areas. Seven women were pregnant. The most affected age group was between 16 and 45 years old (601 cases; 50.9%). Admixed ethnicity was the most recorded (80.1%). A total of 75.4% of the patients had up to four years of study. A proportion of 39.7% of the bites were related to work activities. Maintenance and repair services were the most cited by patients with formal occupations (46.4%), followed by agricultural and forestry activities (45.6%). Most bites occurred in the upper limbs (48.8%). Clinico-epidemiological diagnosis of phoneutrism was done in 169 cases (14.3%), loxoscelism in 156 cases (13.2%) and latrodectism in 19 (1.6%). In 837 patients (70.9%), the agent was not identified. Antivenom was prescribed to 39.0% of the patients. Regarding time elapsed from the bite until medical assistance, 36.9% of the cases received treatment within the first hour after the spider bite, 33.0% within 3 hours and 30.1% with more than 3 hours after bite (Table 2).

**Table 2:** Characteristics of the 1,181 spider bites reported in the State of Amazonas, 2007 to 2014.

Characteristics (completeness)	Number	%
**Sex (n = 1,181; 100%)**		
Male	777	65.8
Female	404	34.2
**Area of occurrence (n = 1,159; 98.1%)**		
Rural	690	59.5
Urban	469	40.5
**Age group (in years) (n = 1,181; 93.9%)**		
0–15	220	18.6
16–30	300	25.4
31–45	301	25.5
46–60	214	18.1
≥61	146	12.4
**Ethnicity (n = 1,145; 96.9%)**		
Admixed	916	80.1
White	85	7.4
Indian	108	9.4
Black	31	2.7
Asian	5	0.4
**Education (in years) (n = 648; 54.9%)**		
Illiterate	59	9.1
1–4	430	66.3
5–8	71	11.0
≥8	88	13.6
**Work-related accident (1,060; 89.8%)**		
Yes	421	39.7
No	639	60.3
**Formal occupation (n = 552; 46.7%)**		
Maintenance and repair services	256	46.4
Farmer/Fisher	252	45.6
Industry employee	27	4.9
Other	17	3.1
**Anatomical region of the bite (n = 1,138; 96.4%)**		
Head	37	3.2
Upper limbs	555	48.8
Trunk	36	3.2
Lower limbs	510	44.8
**Probable type of envenoming (n = 1,181; 100%)**		
Phoneutrism	169	14.3
Loxocelism	156	13.2
Latrodectism	19	1.6
Non-identified envenoming	837	70.9
**Antivenom prescription (n = 1,130; 95.7%)**		
Yes	441	39.0
No	689	61.0
**Time elapsed from bite to medical assistance (hrs) (n = 1,112; 94.2%)**		
≤1	410	36.9
1–3	367	33.0
4–6	168	15.1
7–12	58	5.2
13–24	51	4.6
>24	58	5.2

### Spatial distribution

**Fig 2:**
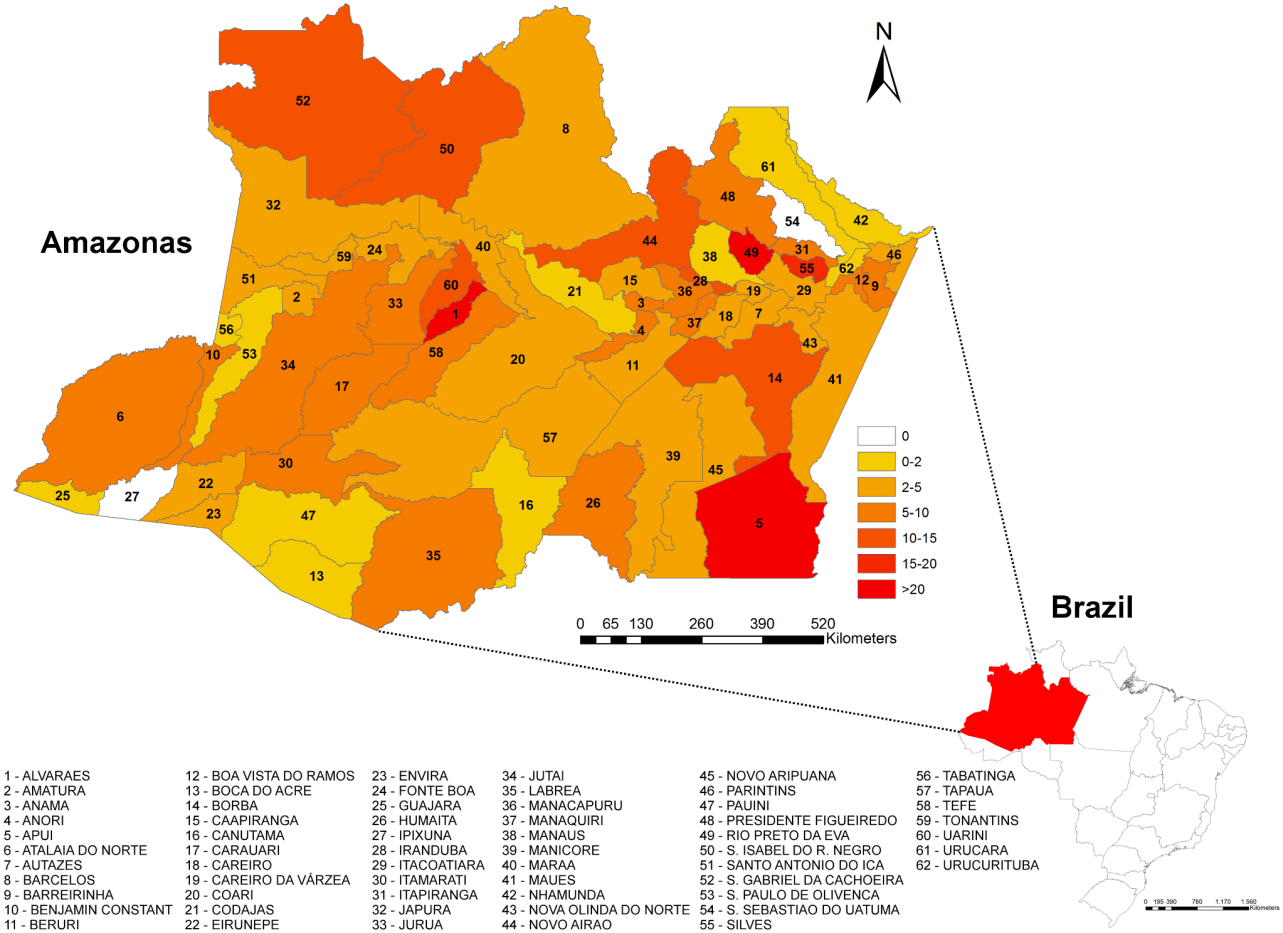
Seasonality of spider bites in the Amazonas State, from 2007 to 2014. A) An increase in the number of spider bites in the Amazonas between June and July is seen for all years. B) A correlation between the absolute number of cases and the altimetric river levels was notable.

Incidence rates were unevenly distributed across the Amazonas State, although there were spider bites cases reported from 60 out 62 municipalities. Mapping showed a large area with high incidence rates extending from São Gabriel da Cachoeira (in the Colombian border) to the Uarini/Alvarães municipalities area and in municipalities surrounding Manaus, namely Novo Airão, Iranduba, Rio Preto da Eva and Silves, where incidence rates were higher than 10 cases per 100,000 inhabitants/year. A third hotspot was located in Apuí, in the Southern region of the state. Municipalities with the the higher anual mean incidence were Rio Preto da Eva (91 cases/100.000 inhabitants), followed by Apuí (58 cases/100.000 inhabitants) (Fig 2).

### Factors associated to spider bites incidence

**Fig 3:**
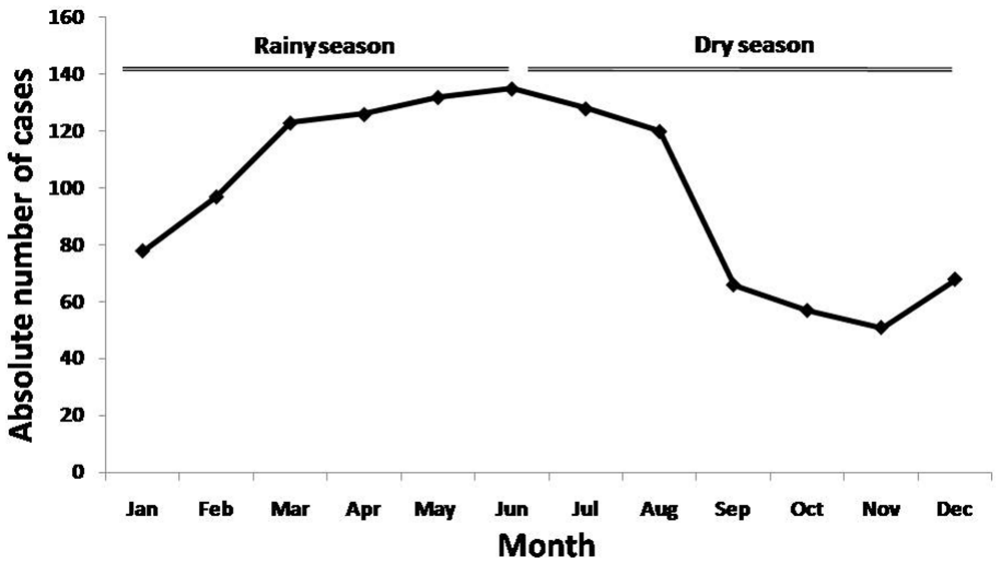
Spatial distribution of spider bites in the State of Amazonas, from 2007 to 2014. Incidence rates were unevenly distributed across the study area. Extensive hot spots are shown from São Gabriel da Cachoeira (in the Colombian border) to the Uarini/Alvarães municipalities area and Manaus surrounding municipalities, with incidence rates >15/100,000/year. The third hotspot is located in Apuí, in the Southern region of the state, with incidence rates >20/100,000/year.

The mean incidence rate was 4 cases per 100,000 inhabitants/year. There was a slight increasing trend during the study period, from 2/ 100,000 inhabitants in 2007 to 6/100,000 in the last three years of study. In rural area the rate was 11.8/100,000/year whereas in urban areas it was 2.1/100,000 /year. There was a gradual increase in the number of spider bites from December to April, which remained high until August (Fig 3).

A multiple linear regression analysis indicated a negative correlation between the spider bites incidence rate and the socioeconomical component poverty rate [Regression coefficient (95% CI) = -0.513 (-0.889 to -0.138); p = 0.008] and the environmental component percentage of areas under the influence of watercourses [Regression coefficient (95% CI) = 0.005 (0.001 to 0.009); p = 0.029] (Table 3).

**Table 3:** Factors associated with spider bites incidence in the State of Amazonas, 2007 to 2014.

Variables	Univariable analysis		Multivariable analysis	
	Regression coefficient (95% CI)	p	Regression coeficiente (95% CI)	p
**Socioeconomical components**				
Distance to capital	-0.002 (-0.0135 | 0.009)	0.725	…	…
Gross municipal product	0.003 (-0.006 | 0.013)	0.472	…	…
MHSPI	3.903 (-2.358 | 10.164)	0.217	…	…
MHSPI access	3.102 (-1.234 | 7.438)	**0.158**	…	…
MHSPI effectiveness	-2.521 (-9.149 | 4.107)	0.450	…	…
MMHDI	42.516 (-21.716 | 106.749)	**0.191**	…	…
EMHDI	22.919 (-18.532 | 64.369)	0.273	…	…
LMHDI	52.686 (-78.509 | 183.882)	0.425	…	…
IMHDI	48.638 (-18.858 | 116.133)	**0.155**	…	…
Poverty rate	**-0.389 (-0.759** | **-0.019)**	**0.040**	**-0.513 (-0.889–0.138)**	**0.008**
Average household income per capita	0.021 (-.0162 | 0.059)	0.260	…	…
Income ratio	-0.001 (-0.004 | 0.003)	0.697	…	…
Rural occupation rate	-0.085 (-0.321 | 0.151)	0.473	…	…
**Enviromental components**				
Percentage of areas under Watercourses influence	**0.003 (0.001** | **0.007)**	**0.206**	**0.005 (0.001–0.009)**	**0.029**
Average deforested area	0.002 (-0.004 | 0.008)	0.412	…	…
Average deforested area increment	0.158 (-0.057 | 0.374)	**0.147**	…	…

CI: Confidence Interval; MHSPI: Mean Health System Performance Index; IDSUS Access: Health System Performance Index Related to Access; IDSUS Effectiveness: Health System Performance Index Related to Effectiveness; MMHDI: Mean Municipal Human Development Index; EMHDI: Educational Munifcipal Human Development Index; LMHDI: Longevity Municipal Human Development Index; IMHDI: Income Municipal Human Development Index.

### Clinical characteristics of spider bites

The most frequent local signs and symptoms observed among spider bite victims were pain (97.3%), edema (65.4%), ecchymosis (6.6%) and pruritus (2.5%) (Table 4). The most frequent local signs and symptoms observed for phoneutrism were pain (97.0%), edema (78.1%), ecchymosis (6.5%) and paresthesia (2.4%). Pain (89.1%), edema (65.4%), ecchymosis (12.2%) and erythema (2.6%) were the most frequent local signs and symptoms for loxoscelism. Pain (100.0%), edema (57.9%) and ecchymosis (5.2%) were the most frequent local signs and symptoms for latrodectism.

**Table 4:** Baseline clinical features of spider bites reported in the State of Amazonas, 2007 to 2014.

Clinical features at admission	Number	%
**Local signs and symptoms (n = 1,083)**		
Pain	1,054	97.3
Edema	708	65.4
Ecchymosis	71	6.6
Pruritus	27	2.5
Erythema	24	2.2
Paresthesia	18	1.7
Necrosis	11	1.0
Blisters	3	0.3
Bleeding	1	0.1
**Systemic manifestations (n = 1,083)**		
Vomiting/diarrhea	40	3.6
Neurological signs (including palpebral ptosis and blurred vision)	39	3.5
Fever/chills	16	1.4
Myolysis/hemolysis (myalgia, anemia, dark urine)	12	1.1
Dizziness	10	0.9
Tremors	6	0.5
Headache	5	0.4
Hemorrhage	4	0.3
Sweating	3	0.3
Renal failure	1	0.1
Hypertension	1	0.1
Dyspnea	1	0.1
Abdominal pain	1	0.1
Sepsis	1	0.1
**Clotting time (n = 465)**		
Normal	383	82.4
Abnormal	82	17.6

Vomiting and/or diarrhea (3.6%), neurological symptoms (3.5%), fever/chills (1.4%) and myolisis/hemolysis (1.1%) were the most frequent systemic manifestations (Table 4), with 4.5% of all cases presenting at least one manifestation at admission. Neurological symptoms (5.9%), vomiting/diarrhea (3.0%) and sweating (1.2%) were observed for phoneutrism. The most frequent systemic manifestations observed for loxoscelism were vomiting/diarrhea (4.5%), neurological symptoms (3.2%) and myolysis/hemolysis (2.6%). Neurological symptoms (5.2%) were the only systemic manifestations observed for latrodectism. Clotting time was abnormal in 17.6% of the cases, and 20.8% in suspected loxoscelism.

Local complications were observed in 1.7% of the patients (Table 5). The most frequent local complications were secondary infection (1.1%), extensive necrosis (0.5%), compartmental syndrome (0.4%) and functional deficit (0.4%). Secondary bacterial infection and functional deficit were observed in 0.6% among phoneutrism cases. For loxoscelism, the most frequent local complications were secondary bacterial infection (3.2%), compartmental syndrome (1.9%), extensive necrosis (1.3%) and functional deficit (1.3%). Secondary bacterial infection occurred in 5.6% of the patients presenting latrodectism. No amputation was recorded. In general, the most frequent systemic complications were acute lung injury (0.5%), shock (0.2%), septicemia (0.1%) and renal failure (0.1%). Phoneutrism was not associated with evolution to systemic complications.

**Table 5:** Clinical complications, severity classification and outcomes of spider bites in patients from the Western Brazilian Amazon, 2007–2014.

Case evolution	Total cases (n = 1,082; 93.2%)	
	Number	%
**Local complications (n = 1,108)**		
Secondary infection	12	1.1
Extensive necrosis	5	0.5
Compartmental syndrome	4	0.4
Functional deficit	4	0.4
**Systemic complications (n = 1,102)**		
Acute lung injury	5	0.5
Shock	2	0.2
Septicemia	1	0.1
Renal failure	1	0.1
**Final case classification (n = 1,144)**		
Mild	871	76.1
Moderate	255	22.3
Severe	18	1.6
**Outcome (n = 1,117)**		
Discharged	1,112	99.6
Death	5	0.4

Most cases were mild (76.1%), followed by moderate (22.3%) and severe (1.6%) cases. There were 5 deaths due to spider bites in the study period (three men and two women, aging from 1 to 61 years old), resulting in a 0.4% lethality rate (Table 5). From the seven pregnant women, four presented severity criteria. Severe cases were more frequent for loxoscelism (3.9%), whereas phoneutrism presented 1.2% and there were no severe cases for latrodectism.

Antivenom was provided to 41.7% of the study population, 33.4% of *Phoneutria* bites and 42% of *Loxosceles* bites. A proportion of 4.6% of moderate and severe spider bite cases received less than the recommended antivenom dosage. A total of 19% of accidents by *Phoneutria* considered moderate received less than the recommended dosage and 100% of severe cases were underdosed. For *Loxosceles* bites, a proportion of 54.9% of moderate cases and 83.3% of severe cases were underdosed. Conversely, 21.2% of the total cases were overdosed and 42.1% of accidents by *Latrodectus* received antivenom inappropriately. Two from five cases (40%) evolving to death did not receive antivenom, one (20%) received underdosed antivenom and two (40%) had no information of antivenom prescrition (Table 6).

**Table 6:** Profile of antivenom administration to 1,180 spider bites patients recorded in the Western Brazilian Amazon, 2007–2014.

Antivenom dosage	Total cases			*Phoneutria* cases			*Loxosceles* cases		
	Mild	Moderate	Severe	Mild	Moderate	Severe	Mild	Moderate	Severe
Underdosage	-	45 (17.6%)	7 (38.9%)	-	8 (19.1%)	2 (100%)	-	28 (62.2%)	5 (83.3%)
As recomended	684 (79.8%)	200 (78.5%)	11 (61.1%)	102 (84.3%)	30 (71.4%)	0 (0%)	80 (86%)	13 (28.9%)	1 (16.7%)
Overdosage	173 (21.2%)	10 (3.9%)	0 (0%)	19 (15.7%)	4 (9.5%)	0 (0%)	13 (14%)	4 (8.9%)	0 (0%)
**Total**	**857 (100%)**	**255 (100%)**	**18 (100%)**	**121 (100%)**	**42 (100%)**	**2 (100%)**	**93 (100%)**	**45 (100%)**	**6 (100%)**

### Factors associated with severity

**Fig 4:**
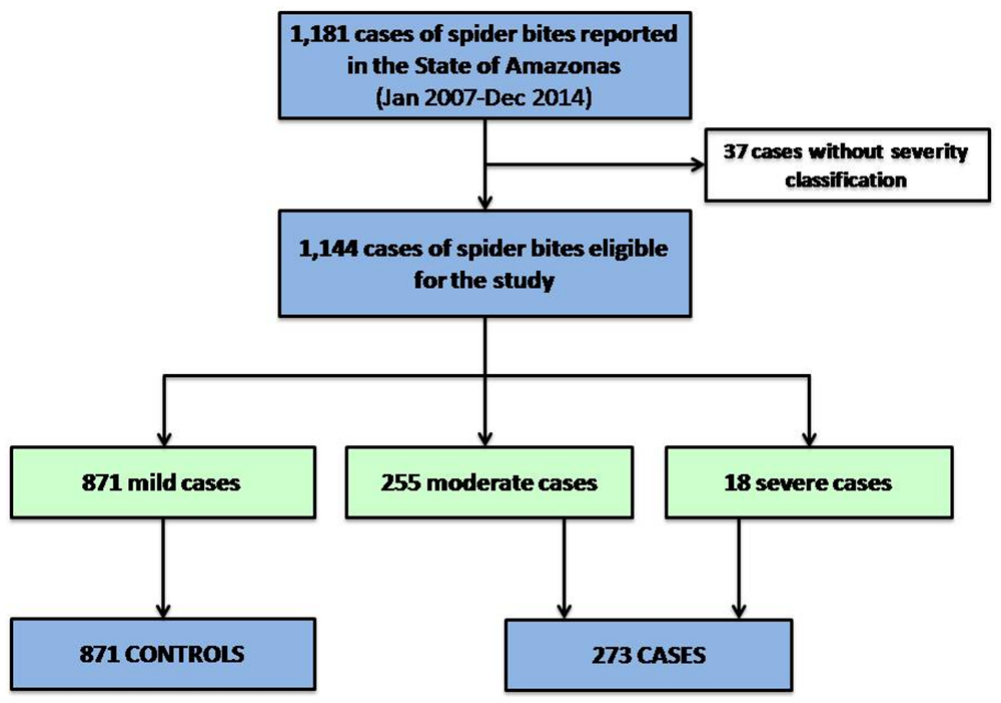
Flow chart of cases and control selection. Selection of cases and controls was based on the Brazilian Ministry of Health classification. All severe and moderate bites were included as cases, whereas mild bites served as controls.

For 273 cases of severe and moderate spider bites during the study period, 872 mild cases served as controls (Fig 4). Table 7 summarizes the results of the logistic regression evaluating independent factors associated with spider bites severity. Envenomings recorded from urban areas [OR = 2.50 (95%CI = 1.41–3.33; p<0.001)], work-related accidents [OR = 2.09 (95%CI = 1.49–2.94; p<0.001)] and indigenous ethnicity [OR = 2.15 (95%CI = 1.19–3.86; p = 0.011)] were independently associated with a risk of developing severity. Living in a municipality located >300 km away from the state capital Manaus [OR = 1.90 (95%CI = 1.28–2.40; p<0.001)] was independently associated with the risk of developing severity and, conversely, living in a municipality with an MHSPI >median [OR = 0.64 (95%CI = 0.39–0.75; p<0.001)] was independently associated with protection from developing severity (Table 8).

**Table 7:** Factors associated with spider bites severity in the State of Amazonas, according to the official surveillance database, 2007 to 2014 (n = 1,180).

Variables	Cases (n)	%	Controls (n)	%	Crude OR (CI 95%)	p	Adjusted OR (CI 95%)	p
**Sex**								
Male	189	69.2	562	64.4	0.81 (0.60–1.08)	0.147	0.92 (0.66–1.30)	0.628
Female	84	30.8	310	35.6				
**Age (years)**								
16–64	206	75.5	638	73.2	1			
≤15	41	15.0	170	19.5	1.34 (0.92–1.95)	0.127	1.13 (0.73–1.76)	0.586
≥65	26	9.5	64	7.3	0.79 (0.49–1.29)	0.350		
**Area of occurrence**								
Rural	194	71.6	465	54.4	**2.13 (1.56–2.86)**	**<0.001**	**2.50 (1.41–3.33)**	**<0.001**
Urban	77	28.4	390	45.6				
**Work related accident**								
Yes	141	56.4	268	34.1	**2.49 (1.87–3.34)**	**<0.001**	**2.09 (1.49–2.94)**	**<0.001**
No	109	43.6	517	65.9				
**Schooling (years of study)**								
≤4	112	56.3	232	54.0	1.10 (0.78–1.54)	0.585	…	…
>4	87	43.7	198	46.0				
**Ethnicity**								
Admixed	216	80.6	680	80.2	1		1	
White	24	8.9	58	6.8	0.77 (0.47–1.27)	0.299	…	…
Indian	18	6.7	84	9.9	1.48 (0.87–2.52)	0.147	**2.15 (1.19–3.86)**	**0.011**
Black	9	3.4	22	2.6	0.78 (0.35–1.71)	0.530	…	…
Asian	1	0.4	4	0.5	1.27 (0.14–11.43)	0.831	…	…
**Anatomical site**								
Head	11	4.0	25	3.0	1		1	
Upper limbs	140	51.5	400	47.9	1.26 (0.60–2.62)	0.542	…	…
Body	4	1.5	31	3.7	3.41 (0.97–12.02)	0.056	1.93 (0.65–5.71)	0.233
Lower limbs	117	43.0	379	45.4	1.42 (0.68–2.98)	0.347	…	…
**Time until medical assistance (hrs)**								
<1	96	36.0	305	37.3	1		1	
1–3	93	34.8	267	32.7	0.90 (0.65–1.26)	0.546	…	…
4–6	45	16.9	116	14.2	0.81 (0.54–1.23)	0.322	…	…
7–12	10	3.7	46	5.6	1.44 (0.70–2.98)	0.315	…	…
13–24	15	5.6	35	4.3	0.73 (0.38–1.40)	0.349	…	…
≥24	8	3.0	48	5.9	1.88 (0.86–4.13)	0.111	1.46 (0.62–3.43)	0.387

OR: Odds Ratio; CI: Confidence Interval.

**Table 8:** Sociodemographical and environmental indicators associated with spider bites severity in the State of Amazonas, 2007 to 2014 (n = 1,180).

Municipality variables	Cases (n)	%	Controls (n)	%	Crude OR (CI 95%)	p	Adjusted OR (CI 95%)	p
**Distance to capital (in km)**								
>300	159	58.2	330	37.9	**2.29 (1.73–3.01)**	**<0.001**	**1.90 (1.28–2.40)**	**<0.001**
**Gross municipal product**								
>Median	178	65.2	669	76.8	**0.56 (0.42–0.76)**	**<0.001**	…	…
**MHSPI**								
>Median	164	60.1	686	78.8	**0.40 (0.30–0.54)**	**<0.001**	**0.64 (0.39–0.75)**	**<0.001**
**MHSPI access**								
>Median	168	61.5	687	78.9	**0.43 (0.32–0.57)**	**<0.001**	…	…
**MHSPI effectiveness**								
>Median	97	35.5	221	25.4	**1.62 (1.21–2.17)**	**0.001**	…	…
**MMHDI**								
>Median	193	70.7	705	80.9	**0.57 (0.42–0.77)**	**<0.001**	…	…
**EMHDI**								
>Median	186	68.1	693	79.6	**0.55 (0.40–0.74)**	**<0.001**	…	…
**LMHDI**								
>Median	161	59.0	667	76.6	**0.44 (0.33–0.59)**	**<0.001**	…	…
**IMHDI**								
>Median	177	64.8	695	79.8	**0.47 (0.35–0.63)**	**<0.001**	…	…
**Average household income per capita**								
>Median	180	65.9	704	80.8	**0.46 (0.34–0.62)**	**<0.001**	…	…
**Income ratio**								
>Median	84	30.8	211	24.2	**1.40 (1.03–1.88)**	**0.031**	…	…
**Unemployment rate**								
>Median	140	51.3	579	66.5	**0.53 (0.40–0.70)**	**<0.001**	…	…
**Rural occupation rate**								
>Median	108	39.6	232	26.6	**1.80 (1.35–2.40)**	<0.**001**	…	…

OR: Odds Ratio; CI: Confidence Interval; MHSPI: Mean Health System Performance Index; IDSUS Access: Health System Performance Index Related to Access; IDSUS Effectiveness: Health System Performance Index Related to Effectiveness; MMHDI: Mean Municipal Human Development Index; EMHDI: Educational Municipal Human Development Index; LMHDI: Longevity Municipal Human Development Index; IMHDI: Income Municipal Human Development Index.

Living in a municipality located >300 km away from the state capital Manaus [OR = 1.53 (95%CI = 1.15–2.02; p = 0.003)] and living in a municipality with an MHSPI <median [OR = 1.91 (95%CI = 1.28–2.47; p = 0.002)] were also independently associated with higher odds of antivenom underdosage. No association was found between time until medical assistance and distance to the capital (p = 0.551).

## Discussion

Official reporting system shows that spider bites prevail across the study area, with a higher incidence in rural areas. A bias in surveillance database could explain the non-plausible negative correlation between the spider bites incidence and the poverty rate, due to the lower odds of riverine and indigenous populations reaching health centers, probably resulting in underreporting. Actually, using estimates of the rate of people bitten by spiders at least once in their lifetime, epidemiological surveillance in rural and indigenous areas would have a sensitivity of only about 10% in the Western Amazon [8]. Importantly, the high proportion of spider bites as a work-related health problem for rural male populations in the Brazilian Amazon highlights the need for public health strategies aiming to reduce these occupational injuries.

Higher spider bites incidence rates were recorded in municipalities surrounding Manaus and in the Southern region of the state, corresponding to areas with intense deforestation. *Phoneutria* and *Loxosceles*, species known to have great potential for adaptation to the man-made environment, were the main causes of bites identified in this study. Bites by *Phoneutria* occur when the spiders are in shoes, piles of sticks or rubbish, or construction material [16]. *Loxosceles* are nocturnal spiders well adapted to the domestic environment, under rocks, wood, or tree bark [23]. There is a marked seasonality of spider bites in the Amazonas state, peaking during the period of heaviest rainfalls. This correlation is possible due to the fact that most rainfall floods the natural habitat of spiders, forcing them to seek new shelters, as observed also for scorpions [24]. The percentage of areas under the influence of watercourses correlates with spider bites incidence, suggesting that altimetric levels could influence the contact between human populatons and venomous spiders. There is evidence of seasonal reproduction for *Phoneutria* in the Amazon, which may affect population abundance [25,26].

The spider bite diagnosis is usually clinical, and definite bites should be based on a clear history of a spider biting the person and then being identified [10]. In this work, only 29.1% of the bites had the causative species identified, among which phoneutrism and loxoscelism prevailed. In general, health services responsible for attending spider bites lack personnel trained in collection and identification of these agents. Identification of spider venom in human tissue has been achieved for *Loxosceles* spp. [27–29] and *Phoneutria nigriventer* [30], but laboratory diagnosis is not available in endemic areas. Some venomous spiders can usually be identified by the general population, which is sufficient for routine management [31]. However, absence of precise information on spider species limits clinical and epidemiological research [11]. In this series, based on epidemiological surveillance data, spider bites mostly caused only minor effects, basically local pain, in agreement with other works [32–37].

*Phoneutria* was the major agent of arachnidism in the Amazonas State based on clinico-epidemiological diagnosis. In South America and Costa Rica, where this genus is found, clinically important bites are mostly reported from Brazil [16,38,39]. Local pain was the major symptom reported by this group of patients (97%). Bites by *Phoneutria* spiders cause immediate local pain of variable severity that radiates proximally up the bitten extremity [10]. Local anaesthetics, opioids, non-steroidal anti-inflammatory drugs and arachnid antivenom have been used empirically for treating *Phoneutria* bites [40,41]. Local edema is less frequent than pain and appeared in 78% of bitten people, in agreement with other estimates in Brazil [39]. In a series of 422 *Phoneutria* confirmed bites almost 90% showed mild envenomation [16]. In this work, 98.8% of cases were classified as mild or moderate envenomation. Severe envenomation was characterized by persistent vomiting and neurological signs such as palpebral ptosis and blurred vision, in agreement with previous studies [16,42]. The major local complications observed were secondary infection and necrosis in *Loxosceles* cases. Necrotic ulcers as suspected spider bites are common in primary care and emergency departments, despite scarcity of evidence for all but *Loxosceles* causing necrotic arachnidism [33]. Although bacterial and fungal causes of necrotic ulcers should always be considered [14,43,44], a proper investigation of *Loxosceles* is needed in endemic areas such as the Amazon.

Systemic envenoming occurred in 4.5% of all cases, with higher frequency among presumed *Loxosceles* cases. Nonspecific symptoms such as vomiting and diarrhea, blurred vision and fever were prevalent within this group of patients, and were the only systemic symptoms recorded from presumed *Phoneutria* and *Latrodectus* cases. Although most cases of systemic loxoscelism are reported in children [15,45–47], in this case series systemic envenoming was reported across all age groups in similar frequencies. The major medical complication of loxoscelism is acute intravascular hemolysis [15,48–51], reported in this study in 1.1% of the patients. Acute renal failure was uncommon, in agreement with previous studies [15,45,52,53].

Although this study showed high frequency of abnormal clotting times in *Loxosceles* patients, only a small proportion evolved with systemic bleeding. Disseminated intravascular coagulopathy (DIC) following *Loxosceles* arachnidism is scarcely reported in the literature [53,54]. The exact frequency and range of clinical manifestations is difficult to establish because most reports of systemic loxoscelism are of presumed rather than confirmed bites. Viscerocutaneous loxoscelism is much less common than the cutaneous form but the frequency varies in different species [1]. In South America, more than 10% of cases of systemic loxoscelism are reported among envenomings with *Loxosceles laeta* [15,45] whereas cases caused by *Loxosceles gaucho* are rare [46].

Local pain was recorded for all patients with suspected *Latrodectus* bites, followed by edema and ecchymosis. Pain is an almost universal feature of latrodectism and can be local bite-site pain, regional or radiating pain, or back, chest, or abdominal pain [10]. In Australia, local pain radiating up the bitten limb or from the bite site is typical [11,55], whereas in North and South America back and abdominal pain predominate [56,57]. In Bahia State, Brazil, abdominal pain was seen in 17% of the patients [37], which was absent in this case series. In this work, no severe *Latrodectus* cases were reported, with only one patient evolving with blurred vision as a neurological symptom. In Manaus, Brazilian Amazon, one suspected case of *Latrodectus* bite, diagnosed only based on clinico-epidemiological criteria, presented headache, abdominal pain, vomiting, muscle spasms, fever, shivering, intense sweating and a significant palpebral edema [58].

Antivenom underdosage was very frequent in this study, namely for *Loxosceles* bites, possibly contributing to severity. Given the scarcity of data on the pharmacoepidemiology of spider antivenoms from endemic areas, antivenom usage prevalences and determinants of beneficial and adverse drug effects are poorly known. In Brazil, antivenom was prescribed in frequencies ranging from 21% in Bahia State [37] to 47% in the South [45]. In this work, antivenom was administered to 41.7% of the study population, 33.4% for *Phoneutria* bites and 42% for *Loxosceles* bites. In Brazil antivenom is less used to treat *Phoneutria* envenomation but is used widely to treat *Loxosceles* envenomation [10]. This study reports that more than 20% of the patients received more than the recommended dosage of antivenom according the Brazilian official guidelines [5], showing that the use of antivenom is still based on clinical discretion, which has led to discrepancies in the proportion of patients treated.

The time it takes to reach health centers from remote isolated areas was hypothesized as a risk factor for spider bite severity in the Brazilian Amazon. However, we found that severe cases were recorded irrespective of the time to medical assistance. Thus, even when timely reaching health services, poor availability of effective care in the Amazon remains a significant barrier for treatment initiation in order to prevent poor outcomes. In the state, only one tertiary reference center for treatment of spider bites envenoming is located in Manaus, the capital. Envenomings recorded from rural areas, Indigenous status and living in a municipality located >300 km away from Manaus presented higher odds of developing severity. This indicates that the absence of specialized centers to treat spider bites represents a considerable obstacle to the provision of quality care to patients in remote Amazonian areas. Consistently, living in a municipality with a higher performance of the health system is a protective factor for severity. In low income regions, inadequate health services performance is a very widespread problem [59]. Despite the high incidence of injuries from venomous animals, there is a lack of systematic health-worker training on diagnosis, specific therapy, and clinical management of complications. Systematic updating of all relevant diagnosis and treatment guidelines was recommended as a strategy to improve the assistance performance, as reported for snakebites and scorpion stings in the Amazon [60]. However, few interventions have been evaluated with rigorous cost-effectiveness trials, and such studies are urgently needed to guide policy. Furthermore, ministry of health should actively help translate research findings into action to improve health-worker performance, and thereby improve health [59].

Interestingly, spider antivenom severity correlates with antivenom underdosage in remote areas with lower health system performances. Although data on *Loxosceles* antivenom efficacy are conflicting and no placebo-controlled trials in human beings have been undertaken [61,62], studies indicate attenuation in dermonecrotic arachnidism in rabbits [63,64] and in the systemic envenoming in mice [64] and rabbits [65]. For *Phoneutria* antivenoms, antivenom therapy efficacy evidence in humans is still scarcer [10]. A *Phoneutria nigriventer* antivenom prevented envenomation symptoms in guinea pigs [66]. Although most symptomatic *Latrodectus* exposures are minor, *Latrodectus* antivenom is associated with shorter symptom duration [67]; its implementation in clinical routine should be evaluated in areas where this genus presents higher incidences. Moreover, innovation in the logistics of antivenoms distribution is also a priority. Current antivenoms require conservation in adequate facilities (2° to 8°C), which are not always available in these remote settings. In addition, multidisciplinary teams training regarding antivenom administration, side effects management, and case monitoring and surveillance may not be appropriate for indigenous and riverine health services.

In conclusion, i) spider envenomings prevailed across the study area, with a higher incidence in rural areas. This information may help improve control strategies against associated morbidity through increasing effectiveness of antivenoms distribution network and other supportive health services in the Amazon; ii) incidence of spider bites was correlated with the period of heaviest rainfalls and was higher in areas under watercourses influence; iii) spider bites can be painful and lead to local complications but rarely result in life-threatening envenoming; iv) main local complications were dermonecrosis and secondary infection in cases diagnosed as *Loxosceles* bites; v) underdosage was very frequent, namely for *Loxosceles* bites; vi) Envenomings recorded from rural areas, Indigenous status and living in a municipality located >300 km away from the state capital Manaus presented higher risk of developing severity; vii) antivenom underdosage coincides with spider bites severity in remote areas of the state with lower health system performances, possibly contributing to severity. There is an urgent need for programmatic efforts to improve case management and antivenom use in the Amazon.

## Supporting Information

S1 File Study dataset. (XLSX)

S2 File Written permission from the original copyright holder of Fig 1. (PDF)

S3 File Written permission from the original copyright holder of Fig 2. (PDF)
