# Supplementary material for: Low Health System Performance, Indigenous Status and Antivenom Underdosage Correlate with Spider Envenoming Severity in the Remote Brazilian Amazon
Source: PLoS One. 2016 May 26;11(5):e0156386. doi: 10.1371/journal.pone.0156386 (PMC4881914; doi:10.1371/journal.pone.0156386)
Supplement: S3 File — (PDF) [file pone.0156386.s003.pdf]

Written permission to publish under a CC BY license

I grant permission for use of Figure 2 contained in the manuscript entitled "Health System Performance, Indigenous status and antivenom Underdosage correlate with spider envenoming severity in the remote Brazilian Amazon" (PONE-D- 16-06026). I assure that this image was produced by me and understand that they will be published in the Plos One Journal under the Creative Commons Attribution License (CCAL) CC BY 4.0. I am also aware that this license allows unrestricted use and distribution, even commercially, by third parties.

Wuelton Marcelo Monteiro
Farmacêutico - Bioquímico
CRF AM/RR 108[illegible]

____

Wuelton Marcelo Monteiro
FMT-HVD/UEA
